# *Staphylococcus aureus* and biofilms: transmission, threats, and promising strategies in animal husbandry

**DOI:** 10.1186/s40104-024-01007-6

**Published:** 2024-03-13

**Authors:** Mengda Song, Qi Tang, Yakun Ding, Peng Tan, Yucheng Zhang, Tao Wang, Chenlong Zhou, Shenrui Xu, Mengwei Lyu, Yueyu Bai, Xi Ma

**Affiliations:** 1 State Key Laboratory of Animal Nutrition and Feeding, College of Animal Science and Technology, China Agricultural University, Beijing, 100193 China; 2 Key Laboratory of Innovative Utilization of Local Cattle and Sheep Germplasm Resources (Co-Construction by Ministry and Province), Ministry of Agriculture and Rural Affairs, School of Agricultural Sciences, Zhengzhou University, Zhengzhou, 450001 China

**Keywords:** Animal husbandry, Biofilm, Mastitis, Mitigation strategies, *Staphylococcus aureus*

## Abstract

*Staphylococcus aureus* (*S. aureus*) is a common pathogenic bacterium in animal husbandry that can cause diseases such as mastitis, skin infections, arthritis, and other ailments. The formation of biofilms threatens and exacerbates *S. aureus* infection by allowing the bacteria to adhere to pathological areas and livestock product surfaces, thus triggering animal health crises and safety issues with livestock products. To solve this problem, in this review, we provide a brief overview of the harm caused by *S. aureus* and its biofilms on livestock and animal byproducts (meat and dairy products). We also describe the ways in which *S. aureus* spreads in animals and the threats it poses to the livestock industry. The processes and molecular mechanisms involved in biofilm formation are then explained. Finally, we discuss strategies for the removal and eradication of *S. aureus* and biofilms in animal husbandry, including the use of antimicrobial peptides, plant extracts, nanoparticles, phages, and antibodies. These strategies to reduce the spread of *S. aureus* in animal husbandry help maintain livestock health and improve productivity to ensure the ecologically sustainable development of animal husbandry and the safety of livestock products.

## Introduction

**Fig. 1:**
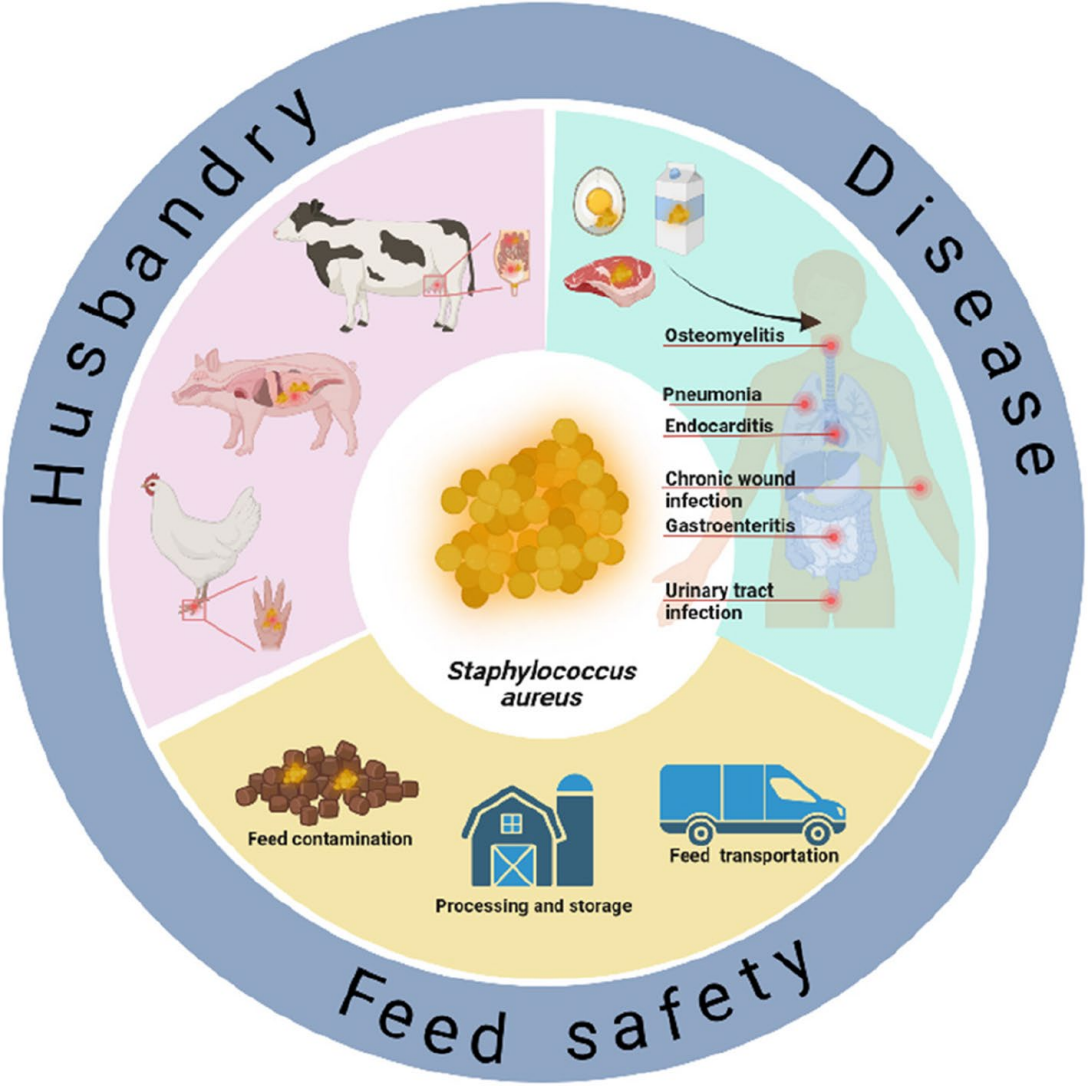
Impacts of *S. aureus* on animal husbandry. These effects range from feed processing, storage, and transportation to livestock diseases and ultimately human health

*Staphylococcus aureus* (*S. aureus*) is a significant foodborne zoonotic pathogen that is responsible for causing diseases in livestock worldwide. *S. aureus* and its biofilms have various implications in livestock and poultry infections, the production and processing of meat products, and the safety of animal feed (Fig. 1). Livestock diseases caused by *S. aureus* are prevalent in pigs [1], cows [2], and poultry [3]. *S. aureus* causes diseases such as mastitis, joint infections, and skin infections in animals [4]. *S. aureus*-induced mastitis in dairy cows is detrimental to the global dairy industry. Moreover, intramammary infections in lactating sheep and goats contribute to economic losses in cheese production [5]. *S. aureus* is also a common cause of infection in broiler chickens and can lead to joint infections such as bacterial chondronecrosis with osteomyelitis [3]. In rabbits, *S. aureus* causes dermal lesions and invades subcutaneous tissues where it can cause pododermatitis, abscess, and mastitis [6]. Additionally, when *S. aureus* infects animals, it forms a biofilm, making it more challenging to eradicate. Biofilm formation promotes the colonization and persistence of *S. aureus* in animals. The antibiotic tolerance of bacteria in biofilms is reportedly 100 to 1,000 times greater than that of planktonic bacteria [7]. Therefore, the formation of biofilms makes infections caused by *S. aureus* more challenging to treat.

*S. aureus* has also been detected in processing chains and meat products. *S. aureus* has been confirmed to be present in air samples, carcasses, and on the surfaces of equipment and tools in slaughter processing lines. During slaughter and processing, *S. aureus* on the skin of animals and slaughterhouse workers can cross-contaminate pork carcasses and pork products [8]. Furthermore, *S. aureus*-induced porcine auricular elephantiasis is common in slaughtered pigs, and this condition increases the risk of *S. aureus* contamination during pork processing [9]. Once *S. aureus* contaminates meat products, it can produce various toxins, such as staphylococcal enterotoxins [10]. Staphylococcal enterotoxins are a class of heat-stable enterotoxins that can induce superantigen activity, leading to immunosuppression and nonspecific T-cell proliferation. Staphylococcal enterotoxins also show resistance to protein hydrolytic enzymes and low pHs, which allows them to remain fully active in the gastrointestinal tract after ingestion.

In recent decades, antibiotics have been employed to improve feed conversion efficiency, treat diseases, and prevent infections in livestock production. In industrialized farming, antibiotics that are frequently used in animal feed to promote growth have led to the development of antimicrobial resistance (AMR) [11]. The development of antibiotic-resistant bacteria further exposes farm workers to new strains of resistant bacteria and increases the risk of infection and illness among these workers [12]. The Ministry of Agriculture and Rural Affairs of the People's Republic of China implemented a policy banning the use of feed antibiotics beginning on July 1, 2020 [13]. This has prompted the feed industry to urgently seek alternatives to feed antibiotics. Currently, novel antimicrobial agents include various options, such as antimicrobial peptides (AMPs), plant extracts, nanoparticles, bacteriophages, and antibodies. AMPs have broad-spectrum antibacterial activity, act rapidly, exhibit good thermal stability and are less likely to induce resistance, making them an ideal alternative to traditional antibiotics. AMPs can also eliminate biofilms through means such as preventing initial bacterial cells attachment, inhibiting biofilm maturation, and eradicating preformed biofilms [14]. Additionally, plant extracts and essential oils have tremendous potential for use as natural preservatives in meat products. Research indicates that due to their extraction from natural sources and biological activities, including antioxidant properties and the inhibition of microbial growth, these products are more easily accepted by consumers as preservatives [15]. Similarly, plant extracts can eliminate *S. aureus* and inhibit the formation of *S. aureus* biofilm. Compared to traditional antibiotic treatments, these new antibacterial agents offer advantages such as safety, environmental friendliness, minimal pollution, few side effects, lower costs, and a reduced likelihood of drug resistance development. Moreover, these materials have significant potential for application in the development of new antibacterial agents for livestock and poultry.

## *S. aureus* and biofilms: transmission and threats in livestock farming

### Transmission pathways

**Fig. 2:**
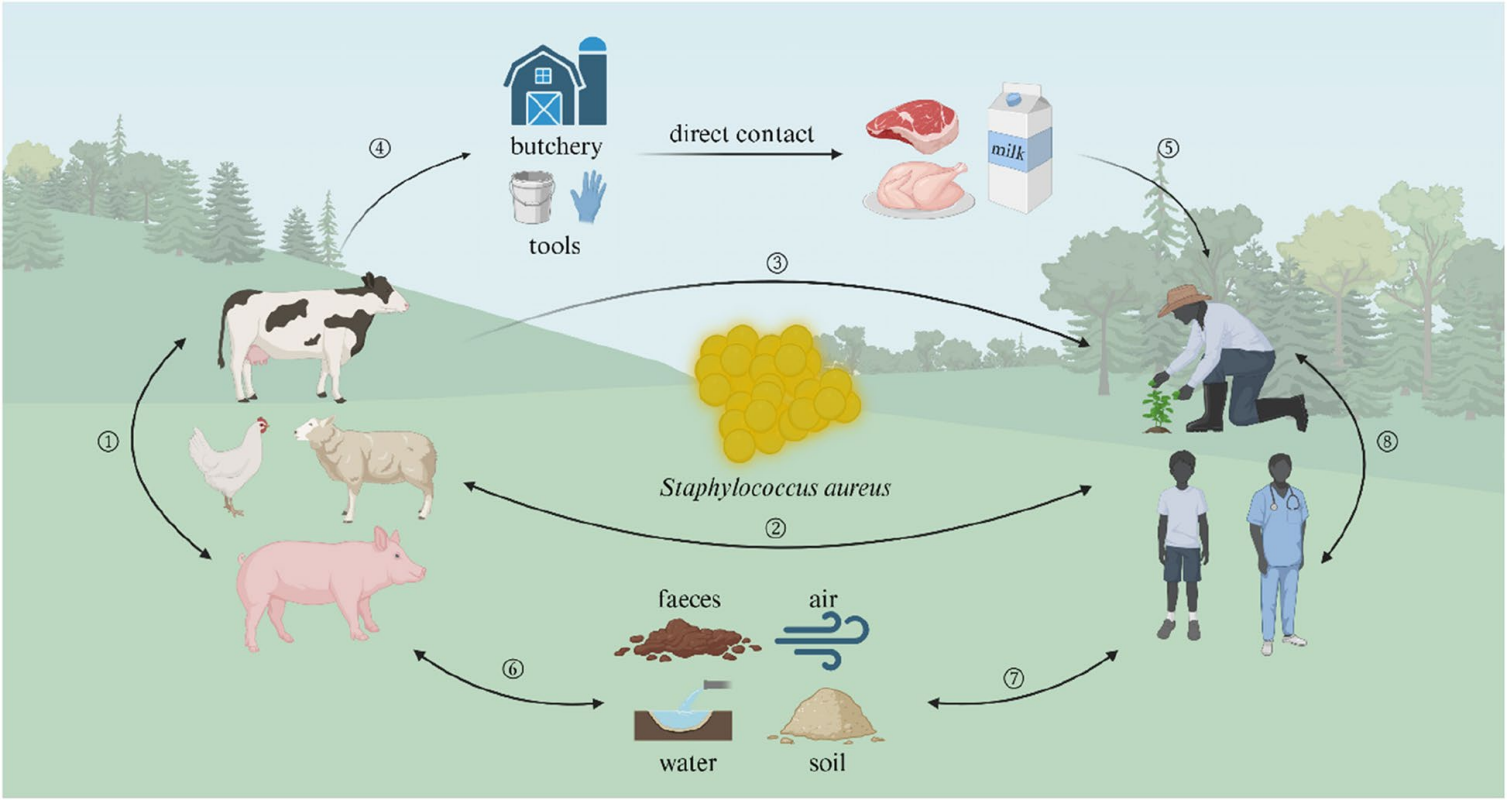
The transmission chain of *S. aureus*. ① Spread in animals by direct contact; ② transmission between humans and animals; ③ transmission by food chains; ④ transmission by processing chains; ⑤ spread to humans through animal products; ⑥ transmission between animals and the environment; ⑦ transmission between humans and the environment; and ⑧ spread among humans

*S. aureus* is commonly found in a wide range of animals, animal-derived products (such as milk and meat), animal-associated environments (contaminated soil, water, and air), and individuals who have close contact with animals, including farm workers, veterinarians, and abattoir workers. During farming activities, *S. aureus* undergoes host switching between different animal species, as well as between humans and animals. The most evident transmission of *S. aureus* occurs through direct contact with the source of infection. The most direct manifestation of infection in noninfected animals occurs after encountering infected animals (Fig. 2 ①). Research has confirmed this transmission pathway, with results showing that the oral inoculation of pigs with *S. aureus* can lead to the infection of previously uninfected pigs [16]. These contact-infected pigs can then transmit the bacterium to new uninfected pigs. Furthermore, the formation of biofilms also facilitates the dissemination of *S. aureus* among animals. In livestock farming, mastitis caused by *S. aureus* is the most common disease. The formation of *S. aureus* biofilms can lead to persistent mastitis infections and promote AMR. Biofilms enable *S. aureus* persistence in the mammary gland and contribute to the spread of mammary infections [17]. In sheep, persistent infections caused by biofilms can result in the loss of mammary glands in ewes or lambs and even lead to death [18]. During breastfeeding, *S. aureus* within biofilms can be shed from the mammary gland, leading to the transmission of mastitis between mothers and infants [19]. In rabbits, the presence of mastitis during lactation and throughout the nursing period leads to the early death of offspring due to mastitis infection [20]. People in close contact with animals, such as farmers and veterinarians, can also be infected with *S. aureus* (Fig. 2 ②). Before 1961, *S. aureus* was believed to be limited to transmission among animals until research revealed that Hungarian cows were the source of *S. aureus* transmitted to their caretakers [21]. This marks the first documented case of *S. aureus* transmission from animals to humans and demonstrated that *S. aureus* can spread horizontally between animals and humans. Later, researchers from various regions around the world discovered that *S. aureus* can spread among different animal species and humans [22]. This population includes species such as pigs, poultry, sheep, goats, and horses.

*S. aureus* can be transmitted through the food chain (Fig. 2 ③). A study reported a food chain transmission event on a mink farm, where the source of infection was feed containing pig offal [23]. This finding indicates that *S. aureus* can spread from pigs to the mink production system, subsequently infecting individuals that come into contact with the mink. Similarly, genomic studies have demonstrated that the prevalence of *S. aureus* in food is attributed to its ability to be transmitted through the food chain via human activities[24]. *S. aureus* can also be transmitted through processing chains and animal products (Fig. 2 ④ and ⑤). *S. aureus* can survive in a biofilm state on both biotic and abiotic surfaces. Due to this characteristic, *S. aureus* can adhere to processing equipment, tools, and environmental surfaces, thus facilitating the spread of *S. aureus* in slaughter and processing chains and the environment [25]. *S. aureus* within biofilms can also contaminate meat products when they come in contact during processing. Animal products such as meat and milk can serve as vectors for the transmission of *S. aureus* from animals to humans. The overall prevalence of *S. aureus* in meat products is 24.5%, with the highest incidence found in beef samples at 33.08% [26]. *S. aureus* has also been isolated from milk samples, suggesting that *S. aureus* can be transmitted through dairy products [27].

*S. aureus* from farm animals can be disseminated into the environment through air and/or faeces, thereby contaminating the soil, water, atmosphere, and even crops both inside and outside the farm (Fig. 2 ⑥). This phenomenon widens the spread and increases the distance of *S. aureus* dissemination. The detection of *S. aureus* in surface and air samples further supports the idea that farm environment can act as a carrier of *S. aureus* [28]. Research has shown that *S. aureus* carried in livestock and poultry faeces can form microbial aerosols that spread between the interior and exterior environments of chicken coops [29]. *S. aureus* can also survive in dust in the air or on surfaces, thereby posing a risk of infection to both animals and humans (Fig. 2 ⑦) [30]. On an Italian farm, researchers separated cows infected with *S. aureus* from uninfected cows. However, new infections were still detected among the uninfected cows [31]. Finally, *S. aureus* can also spread among humans (Fig. 2 ⑧). Research has shown that farm workers infected with *S. aureus* can transmit the bacteria to their family members [23]. Individuals working on a farm or residing in close proximity to a farm are more likely to be colonized by *S. aureus*, which means they may be at risk of infection with *S. aureus.*

AMR is considered a serious threat to livestock. Antibiotics are extensively used in livestock farming for nontherapeutic purposes, such as promoting growth and preventing diseases. Research indicates that antibiotics persist at sublethal concentrations in the gastrointestinal system in livestock and slow the growth of pathogenic bacterial populations [32]. This process exerts selective pressure on pathogenic bacteria in the digestive system of livestock that favours the acquisition and maintenance of antibiotic resistance genes (ARGs) and promotes an increase in the relative abundance of resistant strains. ARGs can rapidly evolve through various mechanisms, including horizontal gene transfer and chromosomal mutation. For example, methicillin resistance arises from the *mecA* gene, which encodes an additional penicillin-binding protein 2a (PBP2a) [33]. The modified surface protein has a lower affinity for beta-lactam antibiotics, thereby reducing the bactericidal effectiveness of these agents. The *mecA* gene is chromosomally inserted as part of the mobile genetic element staphylococcal cassette chromosome *mec* [34]. Depending on the type of staphylococcal cassette chromosome *mec*, the added DNA can also carry ARGs on integrated plasmids, leading to multidrug resistance. This transmission can occur within the same animal population and can also spread through bacteria in the environment. When these ARGs spread to the surrounding environment, AMR becomes an environmental pollution issue. Soil is considered a reservoir of ARGs. For instance, when antibiotic-resistant bacteria from the gastrointestinal systems of livestock are excreted, ARGs disseminate into the environment, including into the soil and water [35]. Subsequently, the spread of ARGs increases the likelihood of human exposure, particularly for agricultural workers and those living in nearby areas.

### Threats to livestock farming

The ability of *S. aureus* to produce biofilms is considered one of the significant factors contributing to the onset of mastitis. During a mammary gland infection, the formation of biofilms facilitates the adhesion and colonization of *S. aureus* on mammary epithelial cells, allowing it to survive within the host and leading to chronic or persistent mammary infections [36]. Mastitis is the most common disease in dairy industry. Mastitis in cows leads to a decrease in milk yield and quality, as well as an increase in the mortality rate of the cows. Compared to that in cows, mastitis in sheep has a significant economic impact on farmers. This can result in the loss of mammary glands in ewes or lambs and even lead to death [37]. In smaller flocks, the incidence of mastitis in sheep can be high, potentially affecting more than 30%–50% of animals and resulting in up to 70% flock deaths or culling [38]. In the expanding rabbit farming industry in Europe, *S. aureus* infection can lead to mastitis, skin abscess, and sepsis. In reproductive rabbits, mastitis is the most common clinical manifestation of *S. aureus* infection. Mastitis persists throughout the lactation period and affects both primiparous and multiparous female rabbits [39]. Typically, kits die early during lactation after the onset of mastitis, and rabbits that recover may refuse further lactation or mating. Furthermore, *S. aureus* is also a major cause of lameness in poultry and results from bacterial chondronecrosis with osteomyelitis. Owing to the difficulty in accessing food and water, lame broiler chickens can dehydrate and die, thus causing significant losses in the poultry industry [40].

Biofilms are formed when a group of pathogenic bacteria adhere to a surface and secrete extracellular polysaccharide matrix, which serves as a protective barrier against conventional antibiotic treatment and host defences [41]. This enables the transfer of metabolites and ARGs between different species, thereby increasing overall pathogenicity. AMR is a key factor that reduces the effectiveness of treatment with biofilm-related bacterial infections. *S. aureus* develops AMR primarily due to the difficulty of penetrating biofilms, phenotypic changes acquired when bacteria form biofilms, and the secretion of enzymes by bacteria that deactivate antibiotics [42]. *S. aureus* development of AMR poses a significant challenge to the livestock industry. Research has shown that 77.2% of *S. aureus* strains isolated from bovine mastitis patients are resistant to one or more antibiotics. Notably, isolates capable of forming biofilms exhibit stronger resistance to multiple antibiotics, indicating that the formation of biofilms promotes the spread and evolution of resistance and ultimately leads to the development of more resistant strains [43]. Additionally, AMR can impact livestock productivity, resulting in an increase in mortality rates and levels of morbidity among animals. An increase in AMR also diminishes the efficacy of antimicrobial agents in treating livestock, thereby increasing infection rates and promoting the spread of infection. Ultimately, the reduction in livestock production and trade will lead to a surge in prices for different sources of protein, including meat, eggs, and dairy [44]. A sustained increase in AMR is projected to cause an 11% decrease in livestock production by 2050, further exacerbating the economic situation due to losses in animal production.

*S. aureus* is the most common foodborne pathogen found in processing chains and meat products. In the food industry, contamination caused by *S. aureus* biofilms occurs primarily on the surfaces of animal-origin fresh and frozen foods, as well as on equipment in the processing environment [45]. The formation of biofilms on food processing surfaces can increase bacterial resistance to disinfectants. Commercial disinfectants have proven effective against planktonic bacteria but often fail to eradicate bacteria that have formed biofilms [46]. Disinfectants must overcome the physical barrier of the biofilm to effectively eliminate bacteria, as this barrier prevents the disinfectant from reaching deeper layers. Additionally, bacteria within biofilms can detach during processing and cause contamination after encountering food, thus presenting a continuous risk of cross-contamination [47]. These isolated strains can grow rapidly in food under favourable conditions, and they often carry genes encoding enterotoxins. If food is properly cooked, *S. aureus* will be killed. However, under conditions of temperature abuse, *S. aureus* can grow in food, produce heat-stable enterotoxins, and cause food poisoning [48]. Therefore, controlling *S. aureus* infection during animal slaughter and meat processing is crucial for reducing the spread of *S. aureus* and preventing foodborne poisoning.

## *S. aureus*-related infections and biofilm formation in animals

*S. aureus* spontaneously forms biofilms during infection and possesses a remarkable array of virulence factors that are responsible for attachment, colonization, invasion, and evasion of the host immune system. For example, *S. aureus* utilizes adhesins to initiate invasion by attaching to the surface of host cells [49]. After invasion, *S. aureus* induces cytoplasmic and mitochondrial Ca^2+^ overload, resulting in both apoptotic and necrotic cell death [50]. In livestock farming, the formation of biofilms by *S. aureus* can lead to persistent udder infections and antibiotic treatment failure. The presence of biofilm components in mastitis may be associated with the duration and severity of the condition. Biofilms have a complex structure consisting of multiple layers embedded in the extracellular matrix and are primarily composed of polysaccharide intercellular adhesin (PIA) [51]. In addition to PIA, the biofilm matrix is composed of various microbial surface components recognizing adhesive matrix molecules (MSCRAMMs), such as fibronectin-binding proteins (FnBPs), clumping factor A, and protein A, which facilitate bacterial adhesion to host cells and initiate biofilm formation [52]. Although studies have shown the presence of biofilm components such as mucus and PIA in mastitis samples, the mechanism of *S. aureus* biofilm formation within the mammary gland has not been confirmed [53]. Additionally, *S. aureus* can cause mastitis in different stages of pregnancy, and infection often persists into the lactation period. *S. aureus* persists during the nonlactating period in cows because the formation of biofilms can facilitate the effective adhesion of *S. aureus* to epithelial cells of the mammary gland [54]. Therefore, maintaining udder cleanliness and adhering to regular milking practices contribute to protecting the health of cows from infection, thus reducing the risk of infection.

**Fig. 3:**
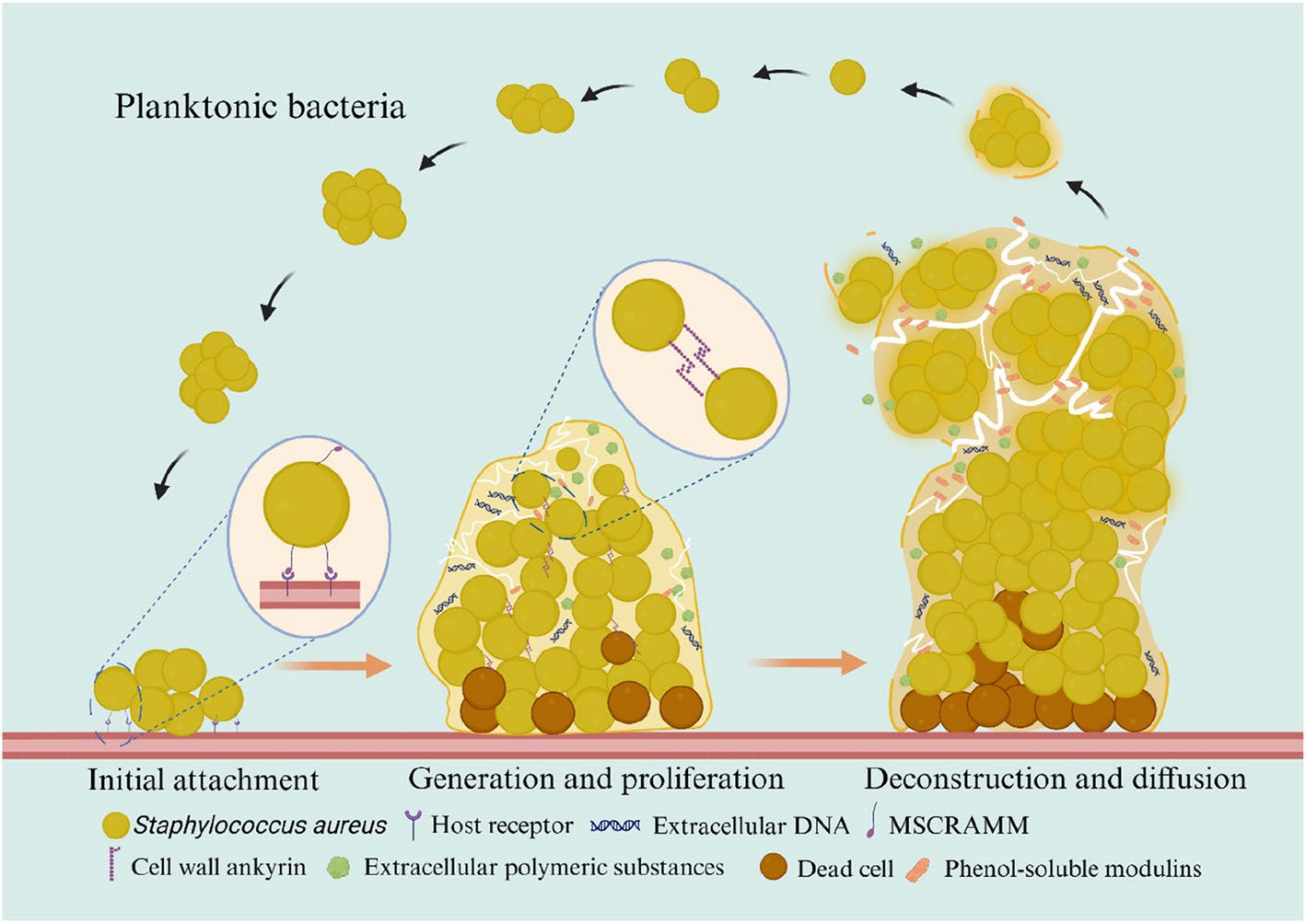
The process of *S. aureus* biofilm formation. Previous reports have classified the biofilm formation process into several stages including initial attachment, proliferative growth, and deconstruction and diffusion. The attachment phase is subdivided into reversible and irreversible attachment, with free *S. aureus* first reversibly attaching to the surface of inert or active entities and then forming irreversible attachments by secreting extracellular substances such as proteins, polysaccharides, lipids, DNA, and other substances. Then, *S. aureus* further expands the scale of the biofilm through polysaccharide-dependent and polysaccharide-independent pathways. In addition, the binding of associated surface proteins results in tighter binding of adjacent cells. Finally, the mature tower biofilm diffuses through various extracellular polymeric substance cracking mechanisms

In the following sections, we will provide a detailed description of the process of *S. aureus* biofilm formation to establish a theoretical foundation for the development of therapeutic strategies for treating *S. aureus* infections in the livestock and poultry industry. The development of *S. aureus* biofilms is tightly controlled by a complex global regulatory system that involves the regulation of numerous related proteins and can be divided into three primary stages: (1) initial attachment, (2) extracellular matrix generation and cell proliferation, and (3) biofilm deconstruction and bacterial dispersal (Fig. 3).

### Initial attachment

Initial attachment is the first stage in which *S. aureus* infects a host to form a biofilm, and planktonic *S. aureus* attaches to biotic or abiotic surfaces. The attachment of *S. aureus* to abiotic surfaces mainly depends on the physical properties of the material plane and the bacterial surface; these factors dictate whether hydrophobic interactions, hydrogen bonds, and electrostatic interactions take place. Binding to biological planes relies on noncovalent interactions between bacterial surface proteins and host matrix proteins [55, 56].

At the initial attachment stage, individual *S. aureus* strains reversibly bind to the surface, and as the bacteria accumulate, they undergo irreversible attachment to the surface. During host infection, *S. aureus* primarily contacts the biologic surface, which is composed of host matrices containing cytokeratin, fibronectin, fibrinogen, and collagen. More than 20 protein modifications on the surface of *S. aureus*, with the C-terminal LPXTG motif, are covalently anchored to the cell wall peptidoglycan by sortase A [57]. The cell wall ankyrin on *S. aureus* peptidoglycan can be divided into multiple groups according to structure and function, and the largest group is a protein family known as MSCRAMMs. This family is characterized by the presence of two IgG-like fold domains arranged in series that bind to ligands through conformational change and promote *S. aureus* attachment to the host biologic surface [52]. The main mechanisms include binding to fibrinogen by a dock-lock-latch mechanism or to collagen by a collagen hug. Abundant MSCRAMMs, which include the fibronectin-binding protein A (FnBPA), the fibronectin-binding protein B (FnBPB), the fibrinogen-binding clumping factors A and B, the serine-aspartate repeat protein family and collagen-binding proteins, have been identified in *S. aureus*.

Attachment to nonbiological surfaces is believed to be facilitated by various physical forces, such as hydrogen bonding and ionic and hydrophobic interactions. Previous research indicated that *S. aureus* exhibited greater adhesion to hydrophobic surfaces than to hydrophilic surfaces [58]. Furthermore, the presence of an autolytic enzyme (AtlA) in *S. aureus* is the main factor responsible for attachment to nonbiological surfaces. AtlA acts as an adhesin and degrades the cell wall, which leads to the release of DNA and in turn promotes the formation of sticky extracellular polymeric substances (EPS) [59]. Moreover, *S. aureus* also has adhesins that does not adhere to the cell wall. The primary adhesins in this category are secreted expanded repertoire adhesive molecules, which include extracellular fibrinogen-binding proteins, extracellular matrix-binding proteins, and extracellular adhesion proteins (Eaps) [55]. Eaps help *S. aureus* adhere to biological surfaces. Moreover, other studies have shown that the extracellular DNA (eDNA) in the *S. aureus* biofilm matrix plays a crucial role in attachment because DNA is negatively charged and bacteria are attached to the host surface by electrostatic interactions [60]. During the attachment stage of biofilm formation, the addition of DNA enzymes could effectively prevent the attachment of *S. aureus*.

### Extracellular polymeric substance generation and cell proliferation

After the irreversible attachment of *S. aureus* was complete, the bacteria produced EPS, which covered the bacterial cells to form microcolonies. Along with the continuous proliferation of bacterial cells, the cells secreted polymer molecules to form a biofilm matrix and finally formed multi-layer accumulated mature biofilms with a certain spatial structure [61]. EPSs are composed of the extracellular polysaccharide intercellular adhesion poly-β(1–6)-*N*-acetylglucosamine (PIA/PNAG), *S. aureus* surface protein G (SasG), teichoic acids, accumulation-associated protein (Aap), and eDNA. *S. aureus* can produce EPS through two mechanisms: polysaccharide-dependent and polysaccharide-independent pathways [62]. The polysaccharide-dependent pathway involves PIA molecules, also known as polymerized *N*-acetyl-glucosamine (PNAG), in EPS. The production of PIA is regulated by the *ica* operon, which consists of 4 genes: *icaA*, *icaD*, *icaB*, and *icaC*. Among these, the *icaA* and *icaD* genes are responsible for encoding and synthesizing the membrane proteins PIA/PNAG, while *icaC* serves as an *O*-succinyl transferase involved in the succinylation of PNAG to modify polysaccharides. *IcaB* is primarily responsible for the deacetylation of proteins. Expression of the *icaABCD* gene promoted the formation of *S. aureus* biofilms [63].

The polysaccharide-independent pathway is generally dependent on protein-mediated intercellular adhesion, biofilm-associated protein (Bap), SasG, FnBPA, and FnBPB. Bap is a macromolecular protein consisting of 2,276 amino acids that is anchored to the cell wall. Under low Ca^2+^ concentrations and acidic conditions, Bap forms a biofilm scaffold for amyloid fibrils, thereby bringing bacteria into contact with neighbouring cells. Bap contributes to the development of *S. aureus* biofilms and promotes adhesion to nonbiological surfaces [64]. SasG and *S. aureus* epidermidis orthologous Aap are sorted enzyme-anchored cell wall proteins that have two domains: the A domain, which mediates bacterial binding to nonbiological surfaces and mammalian epithelial cells, and the B domain, which promotes biofilm formation through protein‒protein interactions. Interestingly, the A domain promotes biofilm formation only after it is hydrolysed by a protease [65]. Aap interacts with AIP to promote biofilm maturation, and fibronectin-binding proteins (FnBPs) are considered key proteins for bacterial invasion into host cells. In addition, cell wall anchors promote the binding of adjacent *S. aureus* cells, which facilitates membrane accumulation [66]. In biofilms, network channels exchange nutrients and waste. These channels are formed by phenol-soluble modulin (PSM) with an α-helical structure. PSMs can disrupt electrostatic or hydrophobic noncovalent interactions between components of the biofilm matrix. Moreover, when the PSM content is high, it leads to lysis of the biofilm.

In addition, the eDNA in *S. aureus* biofilms, which is released from dying cells, is noteworthy [67]. Since the DNA polymer itself carries a negative charge, bacterial adhesion can be enhanced through electrostatic interactions during the attachment phase. Additionally, the EPS component eDNA can induce the expression of ARGs during the biofilm development phase, thus facilitating the transfer of resistance genes between bacteria and increasing biofilm resistance to antibiotics. A previous study showed that the release of eDNA in *S. aureus* was mainly dependent on AtlA. The AtlA mutant exhibited reduced biofilm integrity, and the total biomass of the *S. aureus* biofilm was decreased following the addition of DNase I [68]. The biofilm proliferation stage is often accompanied by the diffusion of “exodus” from the biofilm, which is mediated by nuclease-dependent degradation of eDNA; this stage in biofilm development is tightly regulated [61].

### Biofilm deconstruction and bacterial diffusion

Mature biofilms with uneven tower-like structures were internally filled with water channels that were responsible for the transportation of nutrients, and the differential expression of PSMs formed these fluid channels [69]. As there are more bacteria in the membrane, more PSMs are secreted, and excessive PSM can disrupt the integrity of the biofilm [70]. The final stage of biofilm development is the diffusion of structural bacteria of the biofilm into the environment; this process helps *S. aureus* achieve bacterial diffusion, cell survival, and disease transmission. The basic mechanism of biofilm degradation occurs through the degradation of various components of EPS. *S. aureus* has been found to secrete and produce 10 known proteases, including 7 serine proteases, 1 metalloprotease, and 2 cysteine proteases [71]. Serine proteases degrade FnBPs, and metalloproteases degrade Bap. The protein targeted by cysteine proteases has not been determined. In addition, there is also PSM, which is a surfactant that disrupts molecular interactions within the biofilm matrix and leads to biofilm dispersion. The secretion of these proteases is regulated by the *S. aureus* quorum sensing system auxiliary gene regulation (Agr), which is a peptide-based quorum sensing system. The cell density is sensed by the autoinducing peptide (AIP) [72]. During the development of *S. aureus* biofilms, bacteria in the membrane continuously produce AIP, which accumulates in the matrix. When the extracellular concentration of AIP reaches the threshold, it binds to the histidine kinase AgrC, causing autophosphorylation. This, in turn, induces the expression of RNAIII from the P3 promoter, thereby regulating the expression of hundreds of downstream genes, including those associated with biofilm formation and other virulence factors [73]. In addition, nuclease secretion of EPS component eDNA by *S. aureus* also leads to the separation of biofilms. *S. aureus* secretes two nucleases, Nuc1 and Nuc2, and Nuc1 mutation leads to an increase in biofilm formation, but the overexpression of Nuc1 leads to a decrease in biofilm formation, thus indicating that Nuc1 can degrade biofilms. Nuc2 is a membrane-bound nuclease with an extracellular catalytic domain, but its specific effect has not been determined [74].

## Promising strategies against *S. aureus* infection in livestock

### Antimicrobial peptides

Because *S. aureus* can produce biofilms that prevent antibiotics insertion into the membrane, most antibiotics cannot effectively kill these biofilm bacteria [75]. Therefore, developing new antibiofilm drugs to address this situation is necessary. Host defence peptides are an important part of the innate immune system in all organisms [76]. These peptides can exert direct antibacterial effects, target free bacterial cells (referred to as antibacterial peptides), and bind to antibiotic membranes (referred to as antibiofilm peptides), as well as other immunomodulatory activities, including proinflammatory and anti-inflammatory responses, which are important for the indirect killing of bacteria [14, 77]. AMPs are ideal substitutes for antibiotics because of their antibacterial mechanisms, which prevent bacteria from becoming resistant.

**Fig. 4:**
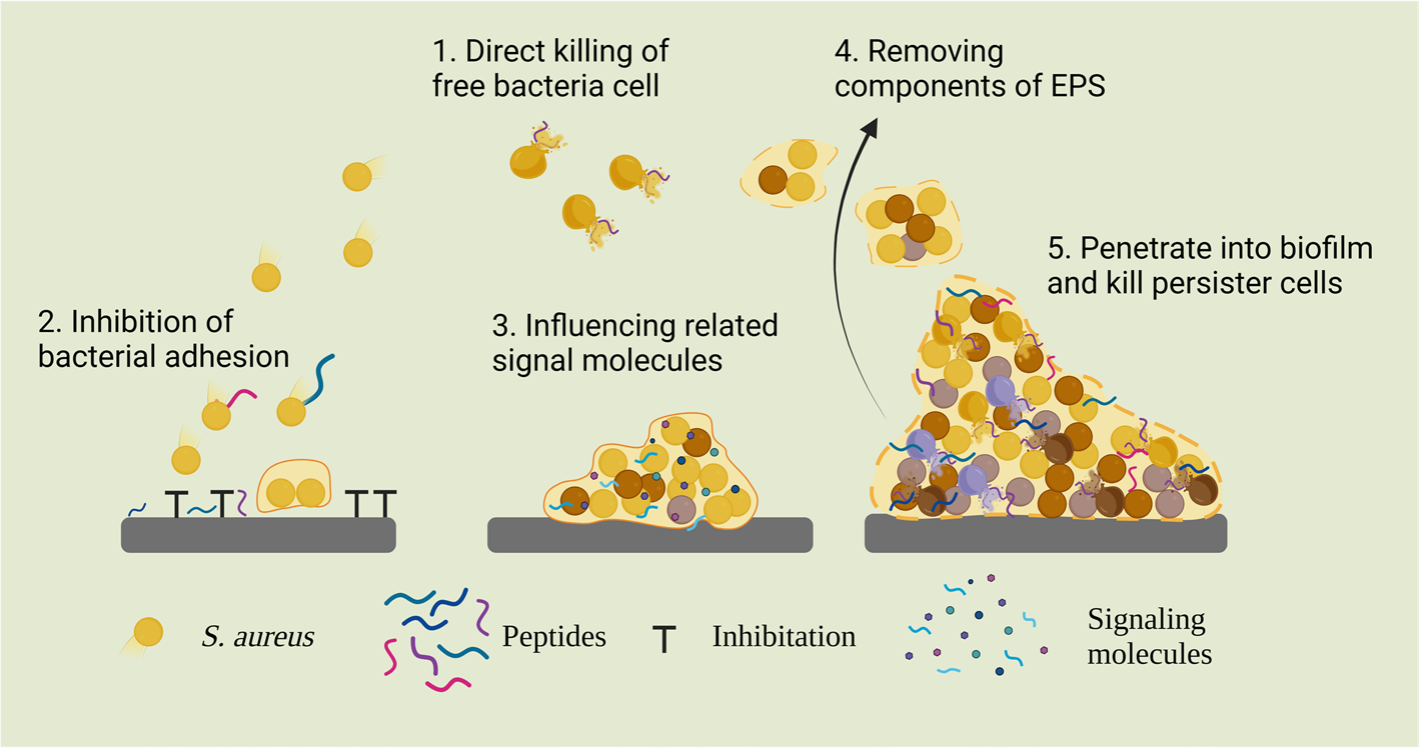
Strategies for the prevention and eradication of biofilms using antimicrobial peptides. The antibiofilm pathways that target the different stages include (1) direct killing of free bacterial cells; (2) inhibition of bacterial adhesion; (3) influencing related signal molecules; (4) removing components of EPS; and (5) penetrating into biofilms and killing cells

AMPs typically employ the following mechanisms to exert antibiofilm activity: (1) directly kill bacteria on free and biofilm surfaces; (2) hinder the initial adhesion of bacteria to biological surfaces; (3) interfere with biofilm formation via related signalling molecules; (4) eliminate components of the EPS from bacterial biofilms; and (5) penetrate into biofilms and kill bacterial cells [78] (Fig. 4).

A recent study reported that the peptide MPX, which was extracted from wasp venom, showed good bactericidal activity and the ability to scavenge biofilms produced by *S. aureus*. Several peptides interfere with biofilm structure by disrupting the biofilm matrix [79, 80]. For example, the fish peptide piscidin3 causes eDNA degradation in EPS, and the amino terminus of piscidin3 binds to Cu^2+^ to enhance DNA cleavage [81]. Studies have demonstrated that the peptide Pm11 exhibits in vitro bacteriostatic activity against common bovine mastitis pathogens, including *Escherichia coli*, *S. aureus*, and *Streptococcus agalactiae* [82]. Wasp venom peptide (Polybia MP 1) belongs to an important class of natural AMP. Polybia MP 1 exhibits antimicrobial activity against multidrug-resistant *S. aureus* isolated from mastitis milk. The peptide achieved bacteriostatic effects by disrupting the inner and outer membranes of bacteria while being nontoxic to mammalian erythrocytes. The results demonstrated the safety and efficacy of the peptide in the treatment of mastitis [83]. Gogoi et al. [84] also isolated methicillin-resistant *S. aureus* from milk and conducted studies to determine the minimum inhibitory concentration, bactericidal kinetics, and cytotoxicity of three forms of the peptide: the linear, dimeric, and tetrameric forms. This peptide possessed the ability to effectively remove MRSA in vitro. Despite the substantial economic losses attributed to mastitis, the therapeutic efficacy of AMPs in dairy models has not been validated. Studies on the clearance of these substances have primarily been limited to in vitro investigations. In recent years, only a few studies have been conducted to validate in vivo therapeutic efficacy of these agents in mouse models of mastitis. The lack of in vivo studies is insufficient to determine the role of AMPs in the treatment of livestock and poultry, and future researchers should combine in vivo and in vitro experiments to increase the persuasiveness of research. Currently, cutting-edge research favours the purposeful and autonomous design and modification of AMPs to achieve therapeutic effects compared to traditional AMP therapy. In a recent study by Field et al. [85], the authors successfully screened three previously uncharacterized nisin derivatives using a site-saturated mutagenesis strategy. These strains exhibited greater inhibitory activity against pathogenic *S. aureus*, including strains associated with bovine mastitis, while demonstrating reduced activity against many of commensal organisms that constitute the milk microbiota, such as *Lactococcus lactis* and *Lactobacillus lactis*. These results suggested that nisin derivatives screened using bioengineering methods are potential novel antimicrobial agents for the treatment of bovine mastitis. The combination of AMP expression systems is at the forefront of current research. Although the current autonomous design of modified AMPs is not yet perfect, its feasibility has been demonstrated. *S. aureus*, one of the most prevalent bacteria found in dermatitis lesions, can induce ongoing infections and inflammation by downregulating the expression of host defence peptides in the skin [86]. Hagfish intestinal peptides effectively inhibited MRSA in vitro and in vivo [87]. Hagfish intestinal peptides reduced bacterial counts and inhibited the secretion of inflammatory cytokines in the lungs or skin of mice with *S. aureus*-induced bacteraemia and skin wound infections. In addition, this peptide binds to bacterial genomic DNA to suppress the expression of the Panton-Valentine leukocidin and nuclease genes, which play major roles in *S. aureus* virulence. Gil et al. [88] designed novel cyclic lipopeptides based on Fusarium analogues. The novel cyclic lipopeptide exhibited antimicrobial activity against MRSA in an in vivo porcine full-thickness wound model, resulting in a reduction in the bacterial count of approximately 3 log CFU/g and a slight increase in wound healing. Another recent study loaded melittin (Mel) into non-ionic surfactant vesicles (NISVs) and confirmed the effective Mel alleviation of skin infections caused by *S. aureus* [89]*.* The NISVs was established for Mel loading (Mel-loaded NISVs) by the thin-film hydration method. Mel-loaded NISVs penetrate the epidermis and dermis to effectively inhibit the growth of bacteria, especially MRSA, in infected skin.

### Plant extracts

Plants have been utilized for the treatment of various diseases since ancient times, and compounds extracted from plants are known for their safety, affordability, and minimal side effects. Certain plant compounds, including flavonoids, alkaloids, terpenoids, phenols, and polyphenols, exhibit antibacterial and antibiofilm activities by inhibiting efflux through pump inhibition and disrupting bacterial quorum-sensing systems. For example, raffinose, which is commonly found in plants, significantly inhibits the formation of *S. aureus* biofilms [90]. Raffinose primarily inhibits the c-di-GMP mechanism of *S. aureus*, and it also inhibits bacterial biofilm formation by interfering with bacterial quorum sensing. This finding suggested that raffinose can serve as a broad-spectrum inhibitor for controlling biofilm formation. Furthermore, several studies have investigated the scavenging effects of plant extracts on common pathogenic bacteria responsible for mastitis [91]. For instance, aloe vera gel extract disrupted the cell membranes of 75% of *S. aureus*, 88% of *Escherichia coli*, and 88% of MRSA strains, resulting in cell lysis [92]. Most available antibiotics are inefficient at eradicating chronic mastitis. To avoid therapeutic failure in *S. aureus* mastitis, new and alternative therapies must be developed in combination with existing antimicrobial agents. Abd El-Hamid et al. [93] investigated the inhibitory effects of essential oils, including carvacrol, linalool, and eugenol, on multidrug-resistant and highly virulent MRSA strains. Linalool was found to have the highest antibacterial and anti-membrane activity, followed by carvacrol and eugenol. In addition, there were synergistic interactions between the essential oils studied and methicillin or vancomycin [93]. Research has indicated that the administration of plant extracts as adjuvants in combination with antibiotics can inhibit the biofilm formation of *S. aureus* [94]. Plant extracts (reserpine, pyrrolidine, quinine, morin and quercetin) have been studied in combination with antibiotics (ciprofloxacin). Overall, these results demonstrate the role of phytochemicals in combination therapies with antibiotics to improve the efficiency of treatments and decrease AMR to antibiotics; these chemicals had substantial effects against both planktonic and biofilm *S. aureus*. In the study by Srichok et al. [95], *Ocimum tenuiflorum* extract had synergistic effects with penicillin or amoxicillin-clavulanic acid against all tested strains, while cefazolin and amikacin had additive effects. In addition, the ideal mode of administration and whether synergistic treatment with plant extracts and antibiotics has therapeutic effects in vivo are still unknown. Garlic extract can affect the growth performance of *Clarias gariepinus* fish and its ability to inhibit invasive *S. aureus* infections [96]. The results showed that the addition of 3.0% garlic extract to fish food improved growth performance, while the addition of 4.5% garlic extract reduced the *S. aureus* bacterial load in fish. Research on the in vivo effects of plant extracts in animals is limited. Researchers need to further explore the therapeutic effects and administration methods of these agents in animals in the future.

### Nanoparticles

In recent years, nanotechnology has introduced new approaches for the treatment of *S. aureus* biofilm-related infections [97]. This is particularly significant as many antibiotics struggle to effectively treat resistant *S. aureus*. Nanoparticles (NPs) have a high surface area-to-volume ratio, which is conducive to enhancing the antibacterial activity of drugs [98]. Moreover, NPs are environmentally friendly and exhibit excellent biocompatibility, rendering them ideal alternatives to antibiotics. Silver has been employed as an antibacterial agent since ancient times, with silver ions demonstrating intrinsic antibacterial activity. Research has shown that silver nanoparticles (AgNPs) exhibit potent bactericidal activity. The antibacterial mechanism is attributed to random physical collisions between AgNPs and bacterial membranes, which, upon penetration into the cytoplasm, result in membrane rupture and bacterial death [99]. Therefore, AgNPs have broad-spectrum antibacterial activity, and AgNPs inhibited up to 98% of *S. aureus* biofilms in vitro. Recent studies have indicated that AgNPs exhibit low toxicity to mammary gland tissue, suggesting that they should not have a detrimental effect on udder tissues [100]. Copper nanoparticles (CuNPs) also have strong antibacterial and antifungal effects. Several studies have demonstrated that silver nanoparticles and copper nanoparticles exhibit inhibitory effects on mastitis pathogens when administered alone or in combination. They have been found to be nontoxic to breast tissue while reducing pathogen viability [101]. Additionally, Ul-Hamid et al. [102] synthesized copper oxide nanoparticles using ginger and garlic root extracts as reducing agents and observed significant inhibition of multidrug-resistant *S. aureus*. Current research on the use of nanoparticles for treating *S. aureus* infections is primarily focused on in vitro validation. In a study aimed at validating the in vivo therapeutic efficacy of nanoparticles, a mastitis model was established in mammals. A concentration of 6.25 μg/mL (25 nm) CuNPs was selected for intramammary treatment in an *S. aureus*-induced mastitis rat model. This concentration was chosen based on the zone of inhibition observed in in vitro sensitivity tests and its minimal cell toxicity in fibroblast lines. In comparison to those in the commercially available antibiotic group, the bacterial load in the CuNP group was lower, the oxidative stress indicators were improved, and the histopathological changes were significantly reversed. These findings demonstrate that CuNPs could serve as a potential alternative for the treatment of bovine mastitis [103]. In addition to mastitis, footpad dermatitis is a prevalent disease in fast-growing broilers. Research has demonstrated that the inclusion of zinc oxide nanoparticles (ZONPs) in the diet can effectively treat MRSA-induced footpad dermatitis. Compared to those in the infected group, the feeding activity and feed conversion efficiency in broilers were significantly greater in the ZONP-treated group than in the infected group, and the pathological changes associated with dermatitis were alleviated [104]. With the expansion of aquaculture worldwide, the demand for functional feeds is continuously increasing, and efforts are underway to improve the efficiency of feed additives. Younus et al. [105] showed that the addition of nanoscale chitosan to feed had an effect on the growth performance and resistance to *S. aureus* infection in silver carp. Chitosan is recommended as a feed additive to improve fish productivity and the immune response to invading pathogens. Interestingly, nanoparticle-loaded disinfectants can overcome the limitations of conventional disinfectants and eradicate *S. aureus* biofilms in poultry farms and slaughterhouses. Complete eradication of *S. aureus* biofilms was observed after treatment with disinfectants loaded with silver and copper nanoparticles at varying concentrations and at different exposure times compared to that after treatment with disinfectants alone [106]. The results demonstrated the potential application of disinfectant nanocomposites for the complete eradication of *S. aureus* biofilms on farms and abattoirs without the development of disinfectant-resistant bacteria. Due to their capacity to disrupt bacterial cell biofilms and penetrate cells and biofilms, nanoparticles can significantly enhance the efficacy of antibacterial agents. Self-assembled chimeric peptide nanoparticles offer several advantages, including a broad antibacterial spectrum, high biocompatibility, and low toxicity, making them excellent alternatives to antibiotics. In some studies, the 14-carbon alkyl chain provides hydrophobicity and self-assembly as the driving force for peptide nanoparticles, while the anti-enzymolysis peptide carries a positive charge to enhance antibacterial activity. These two components are combined to create a nano-chimeric peptide with high stability, excellent antibacterial activity, and strong biocompatibility [107].

### Phages

Another viable approach involves the use of phages, and there are three distinct strategies for biofilm eradication: single phage application, mixed phage application employing two or more phages, and phage-antibiotic combinations. Phages are natural viruses that destroy bacteria, but phage therapy is controversial for several reasons. The advantage of phage therapy for *S. aureus* infections is that it can effectively kill specific bacteria within the membrane, even kill antibiotic-resistant bacteria, without affecting symbionts [108]. Conversely, bacteriophages produce endolysins, which are capable of enzymatically degrading bacterial peptidoglycan, leading to cell lysis and biofilm clearance. A disadvantage of phages is that the phage receptor molecules located on the bacterial cell surface are wrapped within the biofilm matrix so that the phage cannot bind the bacterial cell. Furthermore, bacteria use phage receptors to bind to other bacteria, thus further inhibiting phage attack [109]. Bacteriophages were found to have inhibit *S. aureus* biofilm formation in vitro. For example, the bacteriophages ϕIPLA-RODI and ϕIPLA-C1C reduced *S. aureus* and *S. epidermidis* biofilm formation in vitro [110]. Phage cocktails have also been used to target *S. aureus* biofilms, and Alves et al. [111] demonstrated that the combination of phage K and DRA88 effectively reduced the biomass of *S. aureus* biofilms within 48 h.

Phage therapy has a history of more than 100 years, and its relevance is increasing in response to the growing prevalence of antibiotic-resistant pathogenic bacteria. *S. aureus,* and particularly MRSA, is one of the most extensively researched bacteria for treatment via phage therapy. One potential solution for eradicating MRSA colonization in livestock is phage therapy, which involves the use of bacteriophages; bacteriophages are viruses that infect bacteria [112]. Several studies have investigated phage therapy in farm animals. Drilling et al. [113] conducted two studies involving the use of *S. aureus*-specific phage cocktails in sheep. One study focused on safety and demonstrated that the phage cocktail did not induce inflammatory infiltration or tissue damage when applied to the sinuses of healthy sheep. Another study revealed that phage treatment reduced the number of subepithelial acute inflammatory cells and inhibited biofilm production by *S. aureus* in sheep sinusitis [114]. In an MRSA sinusitis model in piglets, a conflicting result was observed, as phage treatment did not lead to a reduction in bacterial counts, despite the phage cocktail’s effective in vitro killing of MRSA. Another study demonstrated that phage treatment had no adverse effects on pigs. However, this treatment did not lead to a reduction in MRSA levels in patients with MRSA-induced sinusitis. Consequently, the effectiveness of phage treatment for eradicating MRSA from pigs could not be reliably determined [112]. These examples clearly illustrate the need for further studies to comprehensively assess the efficacy of phage treatments in animals. Additionally, phages offer a potential strategy for controlling *S. aureus* infections that cause mastitis. Srujana et al. [115] isolated five phages with lysogenic activity against MRSA. However, notably, these phages exhibited activity primarily in the solid phase, and further efforts may be required to enhance their activity in the liquid phase. Brouillette et al. [116] reported that the StaphLyse™ bacteriophage cocktail had a dose-dependent bactericidal effect against *S. aureus* in vitro. In a mastitis model, a single injection of a phage mixture into the mammary gland significantly reduced the bacterial load of *S. aureus*. Prophylactic use of the phage mixture (4 h pre-challenge) was also therapeutically effective, resulting in a 4 log_10_ CFU reduction per gram of mammary gland. These results further support the potential application of phages as alternatives to antibiotics for controlling *S. aureus*-caused mastitis in dairy cows. Caution is required when applying mouse-based treatment protocols to cows due to differences in their mammary glands. Future studies should explore the feasibility of phage therapy for infected cows, but there are conflicting results in existing studies on *S. aureus* mastitis.

### Antibodies

To develop specific antibodies for the prevention and treatment of *S. aureus* infections, it is important to note that antibodies cannot penetrate bacterial cells. Therefore, antibodies must be designed to target *S. aureus* surface proteins. Studies have attempted to use capsular polysaccharides, aggregation factors A and B, FnBPs, ABC transporters, and amidases as vaccine candidates, as well as clotting factor A, adenosine triphosphate-binding cassette transporters, phosphorus teichoic acid, etc., as potential vaccine antigens [117]. The feasibility of peptidoglycan hydrolase (PGH) as a vaccine was investigated. PGHs are noncovalently linked cell wall-associated enzymes involved in cell wall expansion, cell wall turnover, and daughter cell separation. Autolysin secreted by *S. aureus* is a PGH, and the Atl protein is processed extracellularly to form 62-kDa *N*-acetylmuramyl-L-alanine amidase (Atl-AM) and 51-kDa endo-β-*N*-acetylglucosaminidase. One study revealed that, among all PGHs, Atl-AM is the most antigenic and immunogenic protein; Atl-AM can cause a Th1 immune response to prevent *S. aureus* infection and, furthermore, Atl-AM has a favourable therapeutic effect and is an effective candidate vaccine [118]. In another study, a natural human monoclonal antibody, TRL1068, was discovered using B lymphocyte screening technology. TRL1068 strongly disrupted *S. aureus* biofilms in both in vitro and in vivo experiments. Furthermore, when daptomycin was used in combination with antibiotics, a synergistic effect was observed, which significantly enhanced *S. aureus* sensitivity to antibiotics. This discovery holds great potential for clinical applications [119]. Affinity-purified polyclonal antibodies against the antigen PhnD inhibited *S. aureus* biofilms, and PhnD-deficient strains could not form complete biofilms; furthermore, the addition of PhnD promoted neutrophil phagocytosis of *Staphylococcus* biofilms in vivo. PhnD was found to block the initial attachment and early aggregation of bacteria by introducing antibodies at different stages of biofilm development, and the PhnD antibody intervention strategy may be effective against a variety of *Staphylococci* strains that can form biofilms [120]. Several studies have demonstrated the potential application of antibodies for the treatment of mastitis in dairy cows. Wang et al. [121] constructed an expression vector for scFv-Fc Ab (pcDNA3.1-scFvs-Fc), which was successfully expressed in *E. coli*. The purified antibody underwent in vitro bacteriostatic validation, which revealed its effectiveness in inhibiting *S. aureus* bacterial growth in culture medium. This antibody promoted the phagocytosis of *S. aureus* by peripheral blood neutrophils. Additionally, pcDNA3.1-scFvs-Fc was injected into the mammary glands of mice for expression. The total efficiency of antibody treatment reached 82%. Antibodies, unlike other therapeutic agents, can be validated for therapeutic efficacy using bioengineering methods for expression in hosts and in vivo expression in animals.

## Conclusions

In this review, we introduce the transmission routes and hazards of *S. aureus* and its biofilms and summarize the effective methods for treating *S. aureus* in livestock production that have been studied in recent years. The formation of biofilms increases the duration and incidence of *S. aureus* infection, leading to persistent and recurrent infections. Therefore, inhibiting and eradicating biofilms is a crucial strategy for preventing and treating *S. aureus* infections and has also become a focus for researchers and a research hotspot. In the context of the ban on antibiotic use in feed, several new alternatives have emerged, including AMPs, plant extracts, nanoparticles, phages, and antibodies. However, most of these strategies have been studied in vitro, and there is a lack of in vivo animal studies to clarify their effects and mechanisms of action. Furthermore, the toxicity of these strategies in animals should also be taken into consideration in practice. Therefore, additional in-depth research is needed before these materials can be applied in production. We hope that this review will capture the attention of an increasing number of scientists and contribute to addressing the hazards of *S. aureus* in animal husbandry; future studies will help maintain livestock health and enhance productivity.

## Data Availability

Not applicable.

## Abbreviations

Aap: Accumulation-associated protein
AgNPs: Silver nanoparticles
AIP: Auto-inducing peptide
AMPs: Antimicrobial peptides
AMR: Antimicrobial resistance
ARGs: Antibiotic resistance genes
AtlA: Autolytic enzyme
Atl-AM: *N*-Acetylmuramyl-L-alanine amidase
Bap: Biofilm-associated protein
CuNPs: Copper nanoparticles
Eaps: Extracellular adhesion proteins
eDNA: Extracellular DNA
EPS: Extracellular polymeric substances
FnBPA: Fibronectin-binding proteins A
FnBPB: Fibronectin-binding proteins B
FnBPs: Fibronectin-binding proteins
LA-MRSA: Livestock-associated methicillin-resistant *S. aureus*
Mel: Melittin
MRSA: Methicillin-resistant *S. aureus*
MSCRAMMs: Microbial surface components recognizing adhesive matrix molecules
NISVs: Into non-ionic surfactant vesicles
NPs: Nanoparticles
PGH: Peptidoglycan hydrolase
PIA: Polysaccharide intercellular adhesion
PIA/PNAG: Poly-β(1–6)-*N*-acetylglucosamine
PNAG: Polymerized N-acetyl-glucosamine
PSM: Phenol-soluble regulatory protein
*S. aureus*: *Staphylococcus aureus*
SasG: Surface protein G
ZONPs: Zinc oxide nanoparticles
